# Prolonged Longitudinal Transcutaneous Auricular Vagus Nerve Stimulation Effect on Striatal Functional Connectivity in Patients with Major Depressive Disorder

**DOI:** 10.3390/brainsci12121730

**Published:** 2022-12-17

**Authors:** Shuai Zhang, Jia-Kai He, Gang-Liang Zhong, Yu Wang, Ya-Nan Zhao, Lei Wang, Shao-Yuan Li, Xue Xiao, Zheng-Yi Yang, Bin Zhao, Jin-Ling Zhang, Tian-Zi Jiang, Ji-Liang Fang, Pei-Jing Rong

**Affiliations:** 1 Institute of Acupuncture and Moxibustion, China Academy of Chinese Medical Sciences, Beijing 100700, China; 2 Brainnetome Center, National Laboratory of Pattern Recognition, Institute of Automation, Chinese Academy of Sciences, Beijing 100190, China; 3 Department of Psychiatry, Beijing First Hospital of Integrated Chinese and Western Medicine, Beijing 100026, China; 4 Department of Acupuncture, College of Traditional Chinese Medicine, Southern Medical University, Guangzhou 510515, China; 5 Department of Radiology, Guang’anmen Hospital, China Academy of Chinese Medical Sciences, Beijing 100053, China

**Keywords:** major depressive disorder, prolonged longitudinal transcutaneous auricular vagus nerve stimulation, striatum, cortical striatum circuit, functional connectivity

## Abstract

Background: Transcutaneous auricular vagus nerve stimulation (taVNS) is effective for treating major depressive disorder (MDD). We aimed to explore the modulating effect of prolonged longitudinal taVNS on the striatal subregions’ functional connectivity (FC) in MDD patients. Methods: Sixteen MDD patients were enrolled and treated with taVNS for 8 weeks. Sixteen healthy control subjects (HCs) were recruited without intervention. The resting-state FC (rsFC) based on striatal subregion seed points and the Hamilton Depression Scale (HAMD) were evaluated in the MDD patients and HCs at baseline and after 8 weeks. A two-way ANCOVA test was performed on each rsFC metric to obtain the (group-by-time) interactions. Results: The rsFC values between the left ventral caudate (vCa) and right ventral prefrontal cortex (vPFC), and between the right nucleus accumbens (NAc) and right dorsal medial prefrontal cortex (dmPFC) and ventrolateral prefrontal cortex (vlPFC) are lower in the MDD patients compared to the HCs at baseline, and increase following taVNS; the rsFC values between the left vCa and right, superior occipital gyrus (SOG), and between the left dorsal caudate (dCa) and right cuneus are higher in MDD patients and decrease following taVNS. Conclusions: Prolonged longitudinal taVNS can modulate the striatum rsFC with the prefrontal cortex, occipital cortex, temporal cortex, and intra-striatum, and these changes partly underlie any symptomatic improvements. The results indicate that prolonged longitudinal taVNS may produce beneficial treatment effects by modulating the cortical striatum circuitry in patients with MDD.

## 1. Introduction

Major depressive disorder (MDD) is a global health problem and a common mental disorder in human beings [1,2,3], which presents high morbidity, disability, and mortality rates, and imposes a serious burden on individuals, families, and society in general [2]. Although antidepressants are the first-line treatment for MDD, some MDD patients have failed to benefit from conventional medication therapies because of the low response rate, delayed onset, and various side effects presented in some individuals [4,5,6,7]. Vagus nerve stimulation (VNS) was approved by the FDA for cases of treatment-resistant depression [8], but the risks of surgical intervention, high medical expenses, and potential adverse side effects have greatly limited its application [9,10]. In recent years, transcutaneous auricular vagus nerve stimulation (taVNS) has been demonstrated to be a safe, effective, and noninvasive regulatory technique for the treatment of MDD [11,12]. The treatment can directly stimulate the vagus nerve branch of the ear and produce a clinical effect similar to implanted VNS in terms of reducing depressive symptoms, without the need for surgical intervention [11,13]. The present study explores the antidepressant mechanism of taVNS in the treatment of MDD.

Studies have confirmed that MDD is related to the abnormal structure and function of the cortical–striatal–thalamic–cortical or limbic–cortical–striatal–pallidal–thalamic circuits [14,15,16,17]. The striatum, as the intermediate structure of the circuits mentioned above, receives extensive neuron fiber projections from cortical and subcortical structures [18], and is associated with the areas of reward, emotion, cognition, and motivation. The special function of the striatum makes it a key brain region in the pathogenesis and treatment of MDD. There is increasing evidence that MDD patients have a reduced striatum volume [15,19,20], low excitability levels [21], and low responsiveness to positive stimuli [22,23], which is associated with anhedonia [20]. The excitatory stimulation of the striatal brain region can produce significant antidepressant effects [24,25].

The striatum has a high structural heterogeneity, including the ventral striatum (nucleus accumbens, NAc; ventral caudate nucleus; and ventral putamen) [26] and dorsal striatum (globus pallidus, dorsal caudate nucleus, and dorsal putamen). The former mainly receives fibrous projections from the orbitofrontal cortex, ventromedial prefrontal cortex (vmPFC), and anterior cingulate cortex (ACC) [18,27,28], which also have the roles of emotional regulation and reward functions, while the latter mainly receives fibrous projections from the dorsolateral prefrontal and primary motor cortices [18] that are related to executive and motor functions.

Resting-state functional connectivity (rsFC) plays an important role in explaining the functional connection between brain regions. Functional magnetic resonance imaging (fMRI) studies have shown abnormally high rsFC in the ventral striatum (VS)–dorsomedial prefrontal cortex (dmPFC) [29] and VS–default mode network (DMN) [30], as well as low rsFC in the putamen–ACC and intra-striatum [31] in MDD patients. In particular, the reduced rsFC in the putamen–ACC and intra-striatum have implications with regard to symptomatic monitoring [31]. The other study confirmed that ventral striatum FC could specifically predict the potential risk of adolescent depressive disorders [32]. Therefore, the abovementioned research highlights the importance of cortical striatum circuitry in the pathogenesis of depression.

Increasingly more studies have confirmed that the dysfunction of the striatum and its projection cortical regions are related to MDD, and some scholars have gradually focused on the effect of antidepressant treatments on abnormal rsFC in the striatum with cortical regions. For example, duloxetine could decrease abnormally high rsFC in the right superior VS and putamen subregions with the superior frontal gyrus (SFG), and the reduction in the right superior VS–left SFG rsFC exhibited a greater alleviation of rumination [33]. The increase in the rsFC between the inferior VS and medial prefrontal cortex (mPFC) has been positively related to decreased clinical scores following the treatment of acupuncture plus fluoxetine [34]. Previous studies have also confirmed that instant taVNS increased the rsFC between the left NAc and bilateral mPFC/rACC in the slow-5 band (0.008–0.027), and between the right NAc and occipital gyrus and right lingual/fusiform gyrum in the typical low band (0.008–0.09), and part of them showed a negative association with changes in clinical symptoms [35]. However, no studies have been conducted to examine the effects of prolonged taVNS on the rsFC of MDD patients in the striatum at a subregional level. Therefore, it is of great significance to reveal the seed-to-whole-brain rsFC pattern of striatal subregions to clarify the antidepressant effect of non-drug therapies, such as taVNS.

As a consequence, the purpose of this study is to investigate the therapeutic effect on rsFC changes in striatal subregions after eight weeks of taVNS treatment using a seed-based FC method on medication-free and non-comorbid MDD patients. We selected six striatal subregions (including NAc, caudate nucleus, putamen, and globus pallidus) according to the Brainnetome Atlas [36], and calculated the rsFC between each subregion with the whole brain. In this manuscript, we define “8 weeks of taVNS treatment” as prolonged longitudinal taVNS treatment (for abbreviation purposes, we call this “prolonged taVNS” in the rest of the paper). We hypothesize that prolonged taVNS could modulate the rsFC between striatal subregions and the prefrontal cortex (PFC), and other cortical regions relating to emotional regulation and reward behavior in MDD patients. To verify these hypotheses, we conducted this exploratory study.

## 2. Materials and Methods

This study was a prospective exploratory trial, and was approved by the Ethics Committee of the Institute of Acupuncture and Moxibustion, Chinese Academy of Chinese Medical Sciences, and clinically registered in the National Institutes of Health Clinical Trials Registry (ClinicalTrials.gov ID: NCT03607331, accessed on 31 July 2018). Only the participants who agreed to complete two magnetic resonance imaging (MRI) scans were selected as the research subjects.

### 2.1. Participants

Sixteen patients with MDD were recruited from the Beijing First Hospital of Integrated Chinese and Western Medicine between June 2018 and June 2019. Among them, one case was excluded because of major anxiety. Sixteen HCs matched for gender, age, and educational level were enrolled via advertisements. All the subjects were informed and signed an informed consent form.

The patients who presented with the first onset or recurrence of depression and had not used antidepressants for three months prior to their enrollment were diagnosed by a trained psychiatrist, according to the DSM-5 criteria for MDD. The patients were required to have a current, depressive episode at a mild-to-moderate degree defined by a 17-item Hamilton Depression Scale (17-HAMD) score > 8 and ≤24, a 14-item Hamilton Anxiety Scale (14-HAMA) score < 14, and to not have taken any antidepressants [11]. HCs were required to have both HAMD and HAMA scores < 7, and present no history of psychiatric disorders or psychotropic drug use (Table 1). All the subjects were 18 to 65 years old and right-handed.

The exclusion criteria for all the subjects included a history of serious organic diseases, such as coronary atherosclerotic heart disease, malignancy or renal failure, a history of schizophrenia or other psychiatric disorders, and cognitive or personality disorders. We also excluded subjects who met the DSM-5 criteria for substance-related and addiction disorders experienced within the past six months, presented severe suicidal thoughts or behaviors, were pregnant or breastfeeding, or presented any contra-indications for the MRI scan.

### 2.2. Intervention

**Figure 1:**
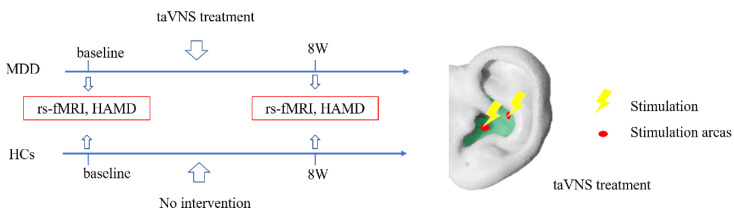
Test flowchart and schematic diagram of taVNS treatment. 8W, 8 weeks; rs-fMRI, resting-state functional MRI; HAMD, Hamilton Depression Scale. Light-green, shaded area represents the auricular concha.

The stimulation points for taVNS (Hwato, SDZ-II B, Suzhou) are located in the auricular concha area, where there is an abundant vagus nerve branch distribution. Both auricles were disinfected with 75% ethanol. Two clips were composed of special silica gel. One clip was fixed on the cymba conchae; the other on the cavum conchae. The dilatational wave was presented at 4/20 Hz (4 Hz for 5 s and 20 Hz for 10 s, alternately). The pulse width was 0.2 ms ± 30% and the intensity was tolerable and without pain for the patients. Bilateral auricles were simultaneously stimulated for 30 min each time, once in the morning and once in the evening. The treatment continued for five days a week, with two days off, for a total of eight weeks. We used a monthly treatment diary to monitor the patients’ treatment status, and all 16 patients completed eight weeks of taVNS treatment with good compliance. The patients received taVNS treatment without the use of any additional therapies during the study period. The HCs received no intervention during the eight-week study. All the subjects completed an assessment of 17-HAMD and resting-state functional MRI (rs-fMRI) scans at baseline and after eight weeks (Figure 1).

### 2.3. MRI Data Acquisition

Imaging data were acquired using a 3T Siemens Skyra MRI scanner with a standard 20-channel head and neck coil. All subjects were requested to keep their eyes closed, not think of anything, stay awake, and remain still without performing head movements during the scan. T1-weighted structural images were acquired with the three-dimensional fast-spoiled gradient-echo sequence to exclude organic lesions in the brain. The following were the parameters of the T1 structural images: repetition time/echo time (TR/TE) = 2530 ms/2.98 ms, flip angle = 7°, scanning field of view (FOV) = 256 mm × 256 mm, spatial resolution = 1 × 1 × 1 mm^3^, and layer thickness/layer spacing = 1.0 mm/1.0 mm. The parameters of the blood oxygen level-dependent (BOLD) gradient echo–echo planar imaging sequence were: TR/TE = 2000 ms/30 ms, flip angle = 90°, FOV = 224 mm × 224 mm, matrix = 64 × 64, number of layers = 32, and layer thickness/layer spacing = 3.5 mm/1.0 mm, 6 min 40 s.

### 2.4. Resting-State fMRI Data Preprocessing

The rs-fMRI data were preprocessed using DPABI (http://rfmri.org/DPABI) [37], which was based on the Statistical Parametric Mapping (SPM12) software in a MATLAB2020a programming environment. In order to guarantee the stability of magnetic resonance signals, the first ten time points were removed and then realign motion correction was performed. All subjects satisfied the head-motion criteria of less than a 1.5 mm maximum translation in any direction of x, y, and z, and a 1.5° rotation in any angular dimension (the framewise displacement (FD) was calculated immediately after each BOLD scan was performed in order to ensure maximum FD < 1.5 mm and 1.5^◦^; otherwise, a rescan was required). White matter, gray matter, and cerebrospinal fluid were segmented using the unified segmentation of high-resolution T1-weighted structural images. The following nuisance covariates (Friston 24 head-motion parameters, polynomial trend and white matter, and cerebrospinal fluid signals) were regressed out. Linear spatial normalization (3 × 3 × 3 mm^3^) was performed in the Montreal Neurological Institute (MNI) space. Spatial smoothing was performed with a 6 mm full-width at a half-maximum (FWHM) Gaussian kernel to improve the signal-to-noise ratio and reduce the negative impact of the registration error. Finally, band-pass temporal filtering (0.01~0.1 Hz) was performed to minimize the effects of low and physiologically high frequencies, such as respiratory and cardiac noise.

### 2.5. Seed Based on FC Analyses

The striatum is divided into twelve subregions in the Brainnetome Atlas (http://atlas.brainnetome.org/) [36], including the bilateral ventral caudate (vCa), dorsal caudate (dCa), globus pallidus (GP), nucleus accumbens (NAc), dorsolateral putamen (dlPu), and ventromedial putamen (vmPu). The seed of striatal subregions was generated using WFU_PickAtlas (Supplementary Figure S1).

The mean time-series of BOLD signals obtained from different striatal subregions was extracted separately, and the mean time-series of the BOLD signals’ correlation coefficients between seeds and all other voxels in the rest of the brain was calculated. A Fisher *r*-to-*z* transformation was carried out to normalize the FC metric.

### 2.6. Statistical Analysis

The demographic characteristics and clinical scale were statistically analyzed using SPSS 26.0 software (SPSS Inc., Chicago, IL, USA) (https://spss.en.softonic.com/). The between-group differences were estimated using an independent sample *t*-test or chi-squared test or Mann–Whitney U-tests, and within-group comparisons were performed by a paired *t*-test.

A two-way mix-ANCOVA was performed on the FC metrics to obtain the (group-by-time) interactions, with gender, age, and FD values as the covariates. A post hoc analysis was conducted by paired *t*-tests for within-group comparisons, and independent sample *t*-tests for between-group differences. A Gaussian random field (GRF) correction was applied to the ANCOVA results (*p* < 0.001 at the voxel level and *p* < 0.05 at the cluster level, two-sided test) and the one-sample *t*-test results (*p* < 0.05 at the voxel level and *p* < 0.05 at the cluster level, two-sided test).

To identify potential FC biomarkers for taVNS in the treatment of MDD and to predict the treatment effects of taVNS, a linear regression analysis controlling for gender, age, and education level was conducted to confirm the relationship between FC values (including FC at baseline and the change in FC at eight weeks vs. baseline) and HAMD improvement (post-treatment vs. pre-treatment). *p* < 0.05 was examined as statistically significant.

## 3. Results

### 3.1. Sample Characteristics

There were no differences in the gender, age, and education level between the MDD and HC groups (all *p* > 0.05). The HAMD score decreased after eight weeks of taVNS treatment in the MDD group (*p* < 0.001) (Table 1).

### 3.2. Within-Group Patterns in Striatum rsFC

As shown in Supplementary Figure S2, the rsFCs in all striatal subregions and their adjacent subcortical regions were significantly enhanced in the HCs and MDD patients. Compared to the HCs at baseline, the rsFC of the MDD patients in the globus pallidus (GP) and dorsolateral putamen with prefrontal cortices and that between the caudate subregions and temporal cortices decreased, and then increased after taVNS treatment. Compared to before treatment, the rsFC of the MDD patients between all striatal subregions and occipital cortices decreased, while the rsFC in the GP and putamen subregions with prefrontal cortices increased after eight weeks of taVNS.

### 3.3. Between-Group Difference in Striatum rsFC

**Figure 2:**
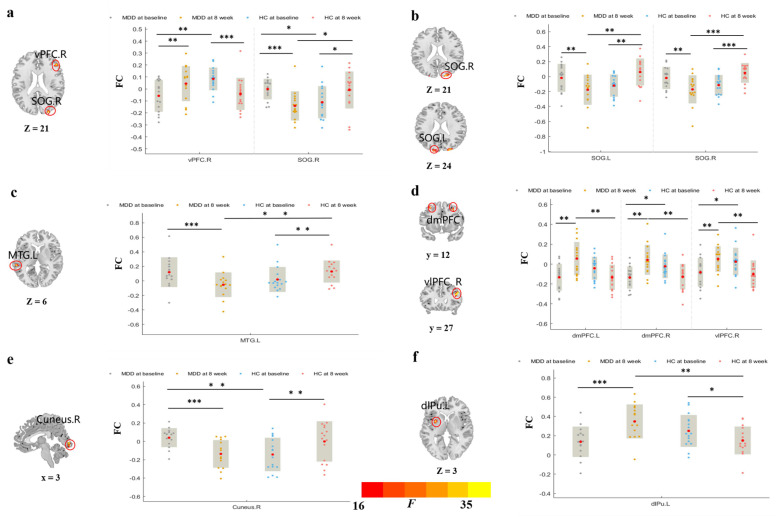
Group × Time interaction on the striatal subregions-based rsFC. (**a**) rsFC of significant Group × Time interaction on the left vCa-right vPFC/SOG and post hoc analysis. (**b**) rsFC of significant Group × Time interaction on the right GP-bilateral SOG and post hoc analysis. (**c**) rsFC of significant Group × Time interaction on the left NAc-left MTG and post hoc analysis. (**d**) rsFC of significant Group × Time interaction on the right NAc-bilateral dmPFC/right vlPFC and post hoc analysis. (**e**) rsFC of significant Group × Time interaction on the left dCa-right cuneus and post hoc analysis. (**f**) rsFC of significant Group × Time interaction on the right dCa-left dlPu and post hoc analysis.vCa, ventral caudate; vPFC, ventral prefrontal cortex; SOG, superior occipital gyrus; GP, globus pallidus; NAc, nucleus accumbens; MTG, middle temporal gyrus; dCa, dorsal caudate; dlPu, dorsolateral putamen; dmPFC, dorsal medial prefrontal cortex; vlPFC, ventrolateral prefrontal cortex. * *p* < 0.05, ** *p* < 0.01, *** *p* < 0.001. *F*, ANOVA statistical value. The bar charts show within- and between-group differences in striatum rsFC. L, Left. R, Right.

Significant group-by-time interactions were observed in the striatal subregions of the rsFC with the prefrontal cortex (PFC), temporal cortex, occipital-cortex areas, and intra-striatal subregions. The results show that taVNS reduces the abnormally higher rsFC behavior between the striatum and temporal and occipital cortices, while it increases the abnormally lower rsFC behavior between the striatum and PFC and intra-striatal subregions (Figure 2, Table 2).

#### 3.3.1. rsFC of Brain Regions Based on vCa

A significant interaction on the left vCa–rsFC was observed in the right ventral prefrontal cortex (vPFC) and right superior occipital gyrus (SOG) (Table 2, Figure 2a).

The left vCa showed lower rsFC with the right vPFC and higher rsFC with the right SOG in the MDD patients compared to the HCs at baseline (right vPFC, *t* = −3.42, *p* = 0.002; right SOG, *t* = 2.70, *p* = 0.011), and were reversed following treatment (right vPFC, *t* = 3.17, *p* = 0.007; right SOG, *t* = −5.0, *p* < 0.001). After eight weeks, the rsFC between the left vCa and right SOG in the MDD patients was reduced compared to the HCs (*t* = −2.64, *p* = 0.013). The HCs had lower rsFC in the left vCa–right vPFC, and higher rsFC in the left vCa–right SOG after eight weeks, compared to the baseline (right vPFC, *t* = −4.38, *p* < 0.001; right SOG, *t* = 2.87, *p* = 0.012) (Figure 2a).

**Figure 3:**
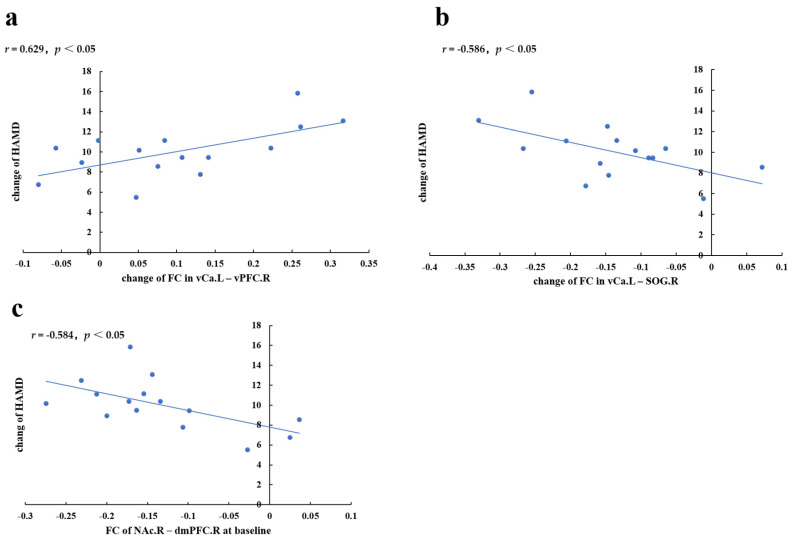
The correlation between the rsFC value and HAMD score. (**a**,**b**) The scatterplot map indicates a correlation between the change in rsFC in the left vCa–right vPFC, left vCa–right SOG, and the remission of the HAMD score. (**c**) The scatterplot map indicates a correlation between rsFC at baseline in R_NAc–R_dmPFC with the remission of HAMD. L, left; R, right. vCa, ventral caudate; vPFC, ventral prefrontal cortex; SOG, superior occipital gyrus; NAc, nucleus accumbens; dmPFC, dorsal medial prefrontal cortex.

We also observed that the change in the HAMD score was positively correlated with the change in the rsFC between the left vCa and right vPFC (*r* = 0.629, *p* = 0.029, Supplementary Table S1, Figure 3a), and was negatively correlated with the change in the rsFC between the left vCa and right SOG (*r* = −0.586, *p* = 0.045, Supplementary Table S1, Figure 3b).

#### 3.3.2. rsFC of Brain Regions Based on GP

A significant interaction on the right GP–rsFC was observed in a bilateral SOG (Table 2, Figure 2b).

After eight weeks, the rsFCs of the MDD patients between the right GP and bilateral SOG decreased compared to the baseline (left SOG, *t* = −3.36, *p* = 0.005; right SOG, *t* = −3.39, *p* = 0.004), and it was also significantly lower than the HCs (left SOG, *t* = −3.46, *p* = 0.002; right SOG, *t* = −3.79, *p* < 0.001), whereas the rsFCs between the right GP and bilateral SOG in the HCs were significantly higher after eight weeks compared to the baseline (left SOG, *t* = 3.72, *p* = 0.002; right SOG, *t* = 4.35, *p* < 0.001) (Figure 2b).

#### 3.3.3. rsFC of Brain Regions Based on NAc

Significant interactions were observed in the bilateral NAc–rsFC. The rsFC of patients with MDD between the left NAc and left middle temporal gyrus (MTG) significantly decreased following treatment (*t* = −4.37, *p* < 0.001), and was lower compared to the HCs at 8 weeks (*t* = −3.15, *p* = 0.004). However, the rsFC present in the left NAc–left MTG of the HCs was significantly higher at eight weeks compared to the baseline (*t* = 1.47, *p* = 0.007) (Table 2, Figure 2c).

The right NAc showed lower rsFC with the right dmPFC and right vlPFC in the MDD patients compared to the HCs at baseline (right dmPFC, *t* = −2.715, *p* < 0.05; right vlPFC, *t* = −2.107, *p* < 0.05), and increased following treatment (left dmPFC, *t* = 4.894, *p* < 0.001; right dmPFC, *t* = 4.93, *p* < 0.001; right vlPFC, *t* = 5.15, *p* < 0.001). After eight weeks, the rsFC of patients with MDD in the right NAc with bilateral dmPFC and right vlPFC was higher compared to the HCs (left dmPFC, *t* = 3.544, *p* = 0.001; right dmPFC, *t* = 3.374, *p* = 0.002; right vlPFC, *Z* = −3.123, *p* = 0.002) (Table 2, Figure 2d).

The change in the HAMD score was negatively related to the rsFC in the right NAc–right dmPFC in the MDD patients at baseline (*r* = −0.584, *p* = 0.046. Supplementary Table S2, Figure 3c).

#### 3.3.4. rsFC of Brain Regions Based on dCa

The rsFC of patients with MDD between the left dCa and right cuneus was higher, in comparison to the HCs at baseline (*t* = 3.47, *p* = 0.002), and significantly decreased following treatment (*t* = −4.19, *p* < 0.001). The rsFC between the left dCa and right cuneus was higher in the HCs at eight weeks, in comparison to the baseline (*t* = 3.41, *p* = 0.004) (Table 2, Figure 2e).

The rsFC of patients with MDD between the right dCa and left dlPu increased following treatment (*t* = 4.76, *p* < 0.001), and it was higher compared to the HCs at eight weeks (*t* = 3.43, *p* = 0.002). However, the rsFC in the right dCa–left dlPu of the HCs was lower at eight weeks, in comparison to the baseline (*t* = −2.71, *p* = 0.016) (Table 2, Figure 2f).

## 4. Discussion

Our study was the first to discuss whether prolonged taVNS impacts seed-based FC in striatal subregions. Consistent with our previous hypothesis, the results indicate that prolonged taVNS can upregulate abnormally low rsFC in the NAc and caudate subregions with PFC and dlPu, and downregulate abnormally high rsFC in the NAc, GP, and caudate subregions with the occipital cortex and MTG areas. Treatment-related rsFC reduces in the left vCa–right SOG, and increases in the left vCa–right PFC, which are specifically related to the relief of depressive symptoms. 

### 4.1. Prolonged taVNS Can Enhance the Intra-Striatal FC of Patients with MDD

Our first important observation was that prolonged taVNS demonstrated a reverse effect on lower intra-striatal rsFC (between the right dCa and left dlPu) in the MDD patients. MDD was characterized by a reduced affective reactivity in the striatum [38]. Relative to the comparison subjects, patients with MDD showed significantly weaker responses to monetary rewarding in the left NAc and caudate bilaterally [39]. However, compared to the HCs, the patients with MDD showed greater activation whilst processing genuine versus posed facial displays of sadness in the caudate and putamen [40]. Therefore, we speculated that the decreased activity of striatum and low intra-striatal rsFC might be the basis of the attenuated reward processing function in the MDD patients. Increasing this activity and rsFC may lead to an increased response to positive stimuli in the MDD patients. The result is similar to the effect of acupuncture combining fluoxetine for MDD, which is significantly increased in the intra-striatal rsFC following treatment relating to the remission of symptoms [34]. Intra-striatal rsFC characterizes the connectivity between striatal subdivisions, which avoids the consideration of the striatum as a single, unified structure [41].

### 4.2. Prolonged taVNS Can Upregulate the rsFC of Patients with MDD in the Frontal–Striatal Reward Network

Prolonged taVNS also demonstrated a normalized effect on the abnormally lower rsFC between striatal subregions (including the left vCa and right NAc) and PFC. The striatum and PFC are important brain regions in the frontal–striatal reward network, and there are afferent and efferent fibrillary projection connections between them [28,42,43]. They are considered to be major brain regions involved in the regulation of motivation, reward, and emotional processes [44,45], which are related to numerous neurological and psychiatric disorders, including MDD [45]. The meta-analysis showed that several frontal–striatal regions participated in reward processing behavior in the MDD patients [46]. The decreased connectivity of the frontal–striatal reward network is considered to be an important cause for the loss of interest, motivation, and happiness (anhedonia) [47].

MPFC is the main brain region used for automated emotional regulation [48,49]. The decrease of rsFC in the NAc–mPFC is related to the abnormal processing of emotions and cognitive behavior [50,51], which is manifested by the lack of reward and increased fear and aversion, whereas the reaction of the NAc and dmPFC to positive stimuli in the MDD patients is reduced [52,53]. LPFC is an important brain region for active emotional regulation [49,54,55]. The activation of vlPFC in the MDD patients is significantly reduced in the regulation of negative emotions [56]. Additionally, the rumination of MDD is usually related to the reduced activation of the left dlPFC and vlPFC [57,58,59].

We could thus think that decreased rsFC in striatal–PFC (including the dmPFC and vlPFC) may underlie the loss of motivation, interest, and pleasure, which seriously affects people with MDD [47]. Enhancing this rsFC may lead to a beneficial influence on the regulation of emotions in MDD patients. Moreover, we observed that increased rsFC in vCa–vlPFC after taVNS was associated with a more significant alleviation of depressive symptoms, and the strength of rsFC in the right NAc–right dmPFC in the MDD patients at baseline was correlated with treatment effects after eight weeks, suggesting that Nac–dmPFC FC may be a distinct biomarker for prolonged taVNS and may predict treatment outcomes for depression.

### 4.3. Prolonged taVNS Downregulates the rsFC of Patients with MDD between the Striatum and Visual and Auditory Areas

Another important observation was the presence of altered striatum rsFC in the visual and auditory cortices; that is, prolonged taVNS could decrease the high rsFC between the striatum (including the NAc, GP, and caudate subregions) and SOG, MTG, and cuneus. The visual and auditory sensory cortices can process information, such as images and sounds from the outside world, causing different psychological or physiological activities. Abnormalities of FC between the striatum and visual areas were also present in insomnia patients, which indicated a disturbed reward sensitivity to sensory and perception stimuli [60]. A number of studies have shown that MDD patients present abnormalities in their cortical structures (including cortical thickness, surface area, and gray-matter volume) in the higher visual cortex, including the fusiform gyrus, superior occipital gyrus, middle occipital gyrus, and cuneus [61,62,63], and also present an abnormal filtering of irrelevant information in the visual cortex from a functional perspective [64]. Other studies also showed that individuals with MDD present increased activity within the visual cortex and striatum to express happiness [65], and a stronger activation whilst processing genuine facial displays of sadness [40]. Moreover, in rat models, reward-stimulating activity elicited a response from the neurons in the primary visual cortex, and the response could accurately predict the timing of the reward [66]. These results indicate that the reward system may regulate visual and auditory perceptions from top to bottom. The innovation of our research was to relate the functional abnormalities of these visual and auditory sensory areas to the core region of the reward system: the striatum. Furthermore, we observed that a greater decrease in the rsFC in the left vCa–right SOG following prolonged taVNS was associated with the alleviation of depressive symptoms. Combined, these results suggest that the striatal–visual circuit may be an objective indicator for the evaluation of clinical responses to taVNS.

In addition, we observed the phenomenon where the changes of rsFC in the HCs were opposed to those in the MDD patients, which was also observed in another resting-state fMRI study [33] and brain structural study [67] in MDD patients, respectively. We consider that the brain function changes dynamically over time [68,69], season or temperature [70], or lifestyle [67], and is also closely related to mental function and body metabolism [69]. Second, changes in rsFC were associated with intervention in MDD patients, but not in HCs. Third, MDD patients are susceptible to the surrounding environment, and the changing trends of brain functions of the two groups over time may or may not be consistent. Future studies should pay attention to the differences over time in the rsFC of the brain in MDD patients and HCs in the non-intervention state. 

### 4.4. Limitation

The advantage of this study was to reveal the modulation of prolonged taVNS on striatum rsFC. However, several issues need to be addressed further. The results were obtained for a small sample of MDD patients, indicating that this study should be considered as an exploratory analysis. To increase the statistical power, replication is required in more MDD patients in future studies. Secondly, despite the HCs used to avoid the effects of the brain’s natural changes over time, we could not exactly determine whether the rsFC changes following treatment were due to a placebo effect, the natural course of the illness, or a taVNS effect. To avoid these effects, a waiting group or a reasonable sham taVNS is necessary in future studies, such as the ear lobe or helix, which is commonly used as a sham taVNS ear site. Thirdly, we used the AAL90 Atlas to define the brain regions that are functionally connected to the striatum, and the brain regions contained relatively large areas and had relatively broad functions. In future studies, we can use the Brainnetome Atlas to define more precise brain regions, in order to provide a more precise neural mechanism for the taVNS treatment of MDD. Fourthly, the untreated HCs generally showed changes in the opposite direction to those shown by the treated MDD participants. The explanation for this finding is not entirely clear and is worthy of further study. Finally, a randomized, large-sample study is needed to confirm the specificity of striatum rsFC changes to prolonged taVNS.

## 5. Conclusions

Our results demonstrate that prolonged taVNS can modulate the rsFC of the cortical striatum circuitry in patients with MDD. The results provide insights into the neural modulation mechanisms of prolonged longitudinal taVNS treatment.

## Figures and Tables

**Table 1:** Characteristics of MDD group and HCs.

Characteristic	MDD (*n* = 15)	HCs (*n* = 16)	*t*/*Z*/*x*^2^	*p* Value
Gender (M/F)	3/12	7/9	1.998	0.157 ^#^
Age (year)	40.9 ± 14.4	35.5 ± 14.8	−0.694	0.488 *
Education (year)	14.3 ± 2.9	14.7 ± 2.2	−0.531	0.595 *
17-HAMD_pre	16.6 ± 4.37	1.19 ± 1.42	4.778	<0.001 ^a^
17-HAMD_aft	6.53 ± 2.45	1.75 ± 1.48	6.634	<0.001 *
HAMD scoreΔ	10.07 ± 3.04	-	12.847	<0.001 ^b^

Note: Data are expressed as the mean ± standard deviation (mean ± SD). Pre: baseline. Aft: 8 weeks. M, male; F, female. Δ, Change in HAMD score; ^#^ *p*, Gender statistics using chi-squared test; * *p*, Age, education, and 17-HAMD_aft statistics using independent sample *t*-test; ^a^ *p*, 17-HAMD_pre, using Mann–Whitney U-tests; ^b^ *p*, MDD at baseline vs. MDD at 8 weeks, using paired *t*-test.

**Table 2:** Anatomical locations of significant Group × Time interaction on the striatal-based rsFC.

Seed Points	Brain Regions	BA	Peak Point MNI Coordinates			Cluster Size (Voxels)	*F* Value
			*x*	*y*	*z*		
Left vCa	Right vPFC	45, 48	48	33	21	15	27.992
	Right SOG	18, 19	27	−93	21	18	27.330
Right GP	Left SOG	18	−16	−93	24	32	26.1023
	Right SOG	18, 19	21	−93	21	46	30.9472
Left NAc	Left MTG	22, 21	−60	−36	6	14	27.518
Right NAc	Left dmPFC	8, 9	−33	12	57	18	23.509
	Right dmPFC	8, 9	30	18	42	26	24.526
	Right vlPFC	44	42	27	24	43	23.927
Left dCa	Right Cuneus	17, 18	3	−90	−3	28	27.938
Right dCa	Left dlPu		−30	3	3	30	35.8427

Note: vCa, ventral caudate; vPFC, ventral prefrontal cortex; SOG, superior occipital gyrus; GP, globus pallidus; NAc, nucleus accumbens; MTG, middle temporal gyrus; dCa, dorsal caudate; dlPu, dorsolateral putamen; dmPFC, dorsal medial prefrontal cortex; vlPFC, ventrolateral prefrontal cortex. BA, Brodmann areas; MNI, Montreal Neurological Institute Space; *F*, statistical value of the peak voxel.

## Data Availability

The data presented in this study are available on request from the corresponding author.

## Supplementary Materials

The following supporting information can be downloaded at: https://www.mdpi.com/article/10.3390/brainsci12121730/s1, Figure S1: Distribution of bilateral Striatum., Figure S2: Within-group patterns of striatal rsFC. Table S1: The correlation between the change of clinical symptoms and change of FC (after_treatment vs. pre_treatment), Table S2: Correlation between changes of clinical symptoms and FC at baseline.
