# Supplementary material for: Prolonged Longitudinal Transcutaneous Auricular Vagus Nerve Stimulation Effect on Striatal Functional Connectivity in Patients with Major Depressive Disorder
Source: Brain Sci. 2022 Dec 17;12(12):1730. doi: 10.3390/brainsci12121730 (PMC9776392; doi:10.3390/brainsci12121730)
Supplement: Supplementary file 1 [file brainsci-12-01730-s001.zip › brainsci-2013638-supplementary.pdf]

Supplementary material

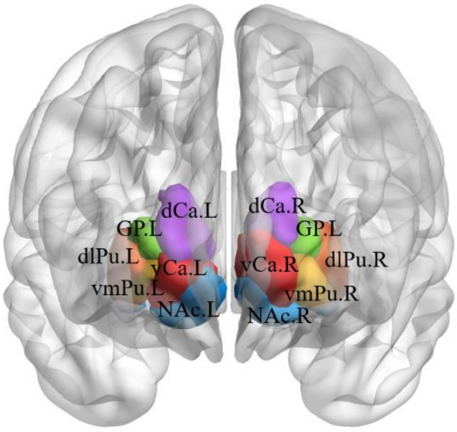

Figure S1. Distribution of bilateral Striatum.

vCa, ventral caudate; dCa, dorsal caudate; GP, globus pallidus; NAc, nucleus accumbens; dlPu, dorsolateral putamen; vmPu, ventromedial putamen. L, left; R, right.

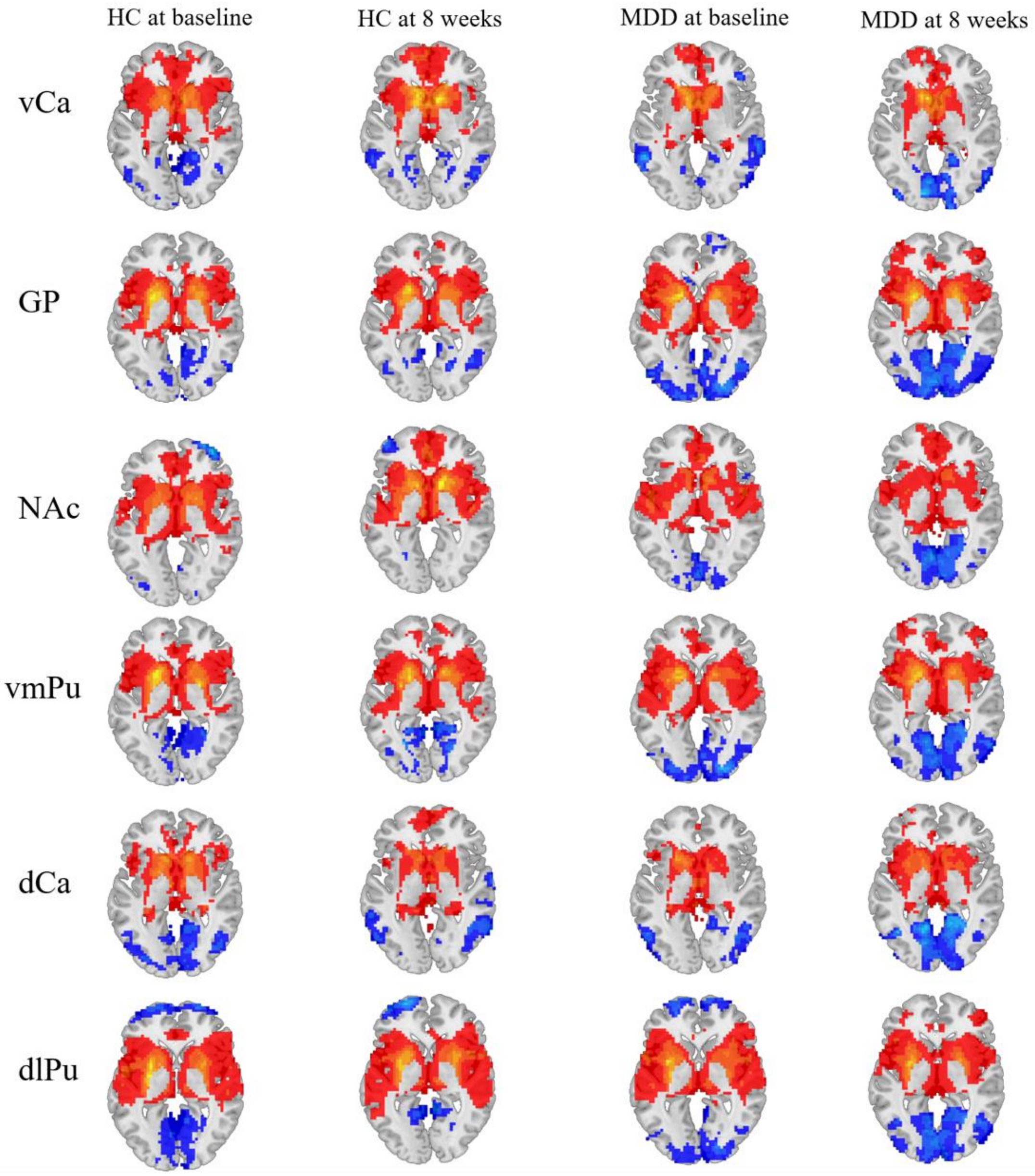

Figure S2. Within-group patterns of striatal rsFC.

vCa, ventral caudate; dCa, dorsal caudate; GP, globus pallidus; NAc, nucleus accumbens; dlPu, dorsolateral putamen; vmPu, ventromedial putamen.

Supplementary Table S1. The correlation between the change of clinical symptoms and change of FC (after_treatment vs. pre_treatment)

Improvement of clinical symptoms	The change of the FC	Relation（*r*）	*p* value
Change of HAMD	vCa.L-vPFC.R	0.629	0.029
	vCa.L-SOG.R	-0.586	0.045
	GP. R-SOG.L	-0.336	0.286
	GP. R-SOG.R	-0.120	0.711
	NAc.L-MTG.L	-0.093	0.774
	NAc.R-dmPFC.L	-0.247	0.439
	NAc.R -dmPFC.R	-0.372	0.234
	NAc.R -vlPFC.R	-0.304	0.337
	dCa.L-Cuneus.R	-0.287	0.365
	dCa.R-dlPu.L	-0.091	0.779

Supplementary Table S2. Correlation between changes of clinical symptoms and FC at baseline

Improvement of clinical symptoms	FC at baseline	*r*	*p* value
Change of HAMD	vCa.L-vPFC.R	-0.336	0.285
	vCa.L-SOG.R	0.518	0.084
	GP. R-SOG.L	0.292	0.357
	GP. R-SOG.R	-0.044	0.893

	NAc.L-MTG.L	0.021	0.948
	NAc.R - dmPFC.L	-0.540	0.070
	NAc.R - dmPFC.R	-0.584	0.046
	NAc.R -vlPFC.R	-0.368	0.239
	dCa.L-Cuneus.R	0.189	0.557
	dCa.R-dlPu.L	0.453	0.140

Note: vCa, ventral caudate; vPFC, ventral prefrontal cortex; SOG, superior occipital gyrus; GP, globus pallidus; NAc, Nucleus accumbens; MTG, middle temporal gyrus; dCa, dorsal caudate; dlPu, dorsolateral putamen; dmPFC, dorsal medial prefrontal cortex; vlPFC, ventrolateral prefrontal cortex.
